# The United States COVID-19 Forecast Hub dataset

**DOI:** 10.1038/s41597-022-01517-w

**Published:** 2022-08-01

**Authors:** Estee Y. Cramer, Yuxin Huang, Yijin Wang, Evan L. Ray, Matthew Cornell, Johannes Bracher, Andrea Brennen, Alvaro J. Castro Rivadeneira, Aaron Gerding, Katie House, Dasuni Jayawardena, Abdul Hannan Kanji, Ayush Khandelwal, Khoa Le, Vidhi Mody, Vrushti Mody, Jarad Niemi, Ariane Stark, Apurv Shah, Nutcha Wattanchit, Martha W. Zorn, Nicholas G. Reich, US COVID-19 Forecast Hub Consortium, Tilmann Gneiting, Anja Mühlemann, Youyang Gu, Yixian Chen, Krishna Chintanippu, Viresh Jivane, Ankita Khurana, Ajay Kumar, Anshul Lakhani, Prakhar Mehrotra, Sujitha Pasumarty, Monika Shrivastav, Jialu You, Nayana Bannur, Ayush Deva, Sansiddh Jain, Mihir Kulkarni, Srujana Merugu, Alpan Raval, Siddhant Shingi, Avtansh Tiwari, Jerome White, Aniruddha Adiga, Benjamin Hurt, Bryan Lewis, Madhav Marathe, Akhil Sai Peddireddy, Przemyslaw Porebski, Srinivasan Venkatramanan, Lijing Wang, Maytal Dahan, Spencer Fox, Kelly Gaither, Michael Lachmann, Lauren Ancel Meyers, James G. Scott, Mauricio Tec, Spencer Woody, Ajitesh Srivastava, Tianjian Xu, Jeffrey C. Cegan, Ian D. Dettwiller, William P. England, Matthew W. Farthing, Glover E. George, Robert H. Hunter, Brandon Lafferty, Igor Linkov, Michael L. Mayo, Matthew D. Parno, Michael A. Rowland, Benjamin D. Trump, Samuel Chen, Stephen V. Faraone, Jonathan Hess, Christopher P. Morley, Asif Salekin, Dongliang Wang, Yanli Zhang-James, Thomas M. Baer, Sabrina M. Corsetti, Marisa C. Eisenberg, Karl Falb, Yitao Huang, Emily T. Martin, Ella McCauley, Robert L. Myers, Tom Schwarz, Graham Casey Gibson, Daniel Sheldon, Liyao Gao, Yian Ma, Dongxia Wu, Rose Yu, Xiaoyong Jin, Yu-Xiang Wang, Xifeng Yan, YangQuan Chen, Lihong Guo, Yanting Zhao, Jinghui Chen, Quanquan Gu, Lingxiao Wang, Pan Xu, Weitong Zhang, Difan Zou, Ishanu Chattopadhyay, Yi Huang, Guoqing Lu, Ruth Pfeiffer, Timothy Sumner, Dongdong Wang, Liqiang Wang, Shunpu Zhang, Zihang Zou, Hannah Biegel, Joceline Lega, Fazle Hussain, Zeina Khan, Frank Van Bussel, Steve McConnell, Stephanie L Guertin, Christopher Hulme-Lowe, V. P. Nagraj, Stephen D. Turner, Benjamín Bejar, Christine Choirat, Antoine Flahault, Ekaterina Krymova, Gavin Lee, Elisa Manetti, Kristen Namigai, Guillaume Obozinski, Tao Sun, Dorina Thanou, Xuegang Ban, Yunfeng Shi, Robert Walraven, Qi-Jun Hong, Axel van de Walle, Michal Ben-Nun, Steven Riley, Pete Riley, James Turtle, Duy Cao, Joseph Galasso, Jae H. Cho, Areum Jo, David DesRoches, Pedro Forli, Bruce Hamory, Ugur Koyluoglu, Christina Kyriakides, Helen Leis, John Milliken, Michael Moloney, James Morgan, Ninad Nirgudkar, Gokce Ozcan, Noah Piwonka, Matt Ravi, Chris Schrader, Elizabeth Shakhnovich, Daniel Siegel, Ryan Spatz, Chris Stiefeling, Barrie Wilkinson, Alexander Wong, Sean Cavany, Guido España, Sean Moore, Rachel Oidtman, Alex Perkins, Julie S. Ivy, Maria E. Mayorga, Jessica Mele, Erik T. Rosenstrom, Julie L. Swann, Andrea Kraus, David Kraus, Jiang Bian, Wei Cao, Zhifeng Gao, Juan Lavista Ferres, Chaozhuo Li, Tie-Yan Liu, Xing Xie, Shun Zhang, Shun Zheng, Matteo Chinazzi, Alessandro Vespignani, Xinyue Xiong, Jessica T. Davis, Kunpeng Mu, Ana Pastore y Piontti, Jackie Baek, Vivek Farias, Andreea Georgescu, Retsef Levi, Deeksha Sinha, Joshua Wilde, Andrew Zheng, Omar Skali Lami, Amine Bennouna, David Nze Ndong, Georgia Perakis, Divya Singhvi, Ioannis Spantidakis, Leann Thayaparan, Asterios Tsiourvas, Shane Weisberg, Ali Jadbabaie, Arnab Sarker, Devavrat Shah, Leo A. Celi, Nicolas D. Penna, Saketh Sundar, Abraham Berlin, Parth D. Gandhi, Thomas McAndrew, Matthew Piriya, Ye Chen, William Hlavacek, Yen Ting Lin, Abhishek Mallela, Ely Miller, Jacob Neumann, Richard Posner, Russ Wolfinger, Lauren Castro, Geoffrey Fairchild, Isaac Michaud, Dave Osthus, Daniel Wolffram, Dean Karlen, Mark J. Panaggio, Matt Kinsey, Luke C. Mullany, Kaitlin Rainwater-Lovett, Lauren Shin, Katharine Tallaksen, Shelby Wilson, Michael Brenner, Marc Coram, Jessie K. Edwards, Keya Joshi, Ellen Klein, Juan Dent Hulse, Kyra H. Grantz, Alison L. Hill, Kathryn Kaminsky, Joshua Kaminsky, Lindsay T. Keegan, Stephen A. Lauer, Elizabeth C. Lee, Joseph C. Lemaitre, Justin Lessler, Hannah R. Meredith, Javier Perez-Saez, Sam Shah, Claire P. Smith, Shaun A. Truelove, Josh Wills, Lauren Gardner, Maximilian Marshall, Kristen Nixon, John C. Burant, Jozef Budzinski, Wen-Hao Chiang, George Mohler, Junyi Gao, Lucas Glass, Cheng Qian, Justin Romberg, Rakshith Sharma, Jeffrey Spaeder, Jimeng Sun, Cao Xiao, Lei Gao, Zhiling Gu, Myungjin Kim, Xinyi Li, Yueying Wang, Guannan Wang, Lily Wang, Shan Yu, Chaman Jain, Sangeeta Bhatia, Pierre Nouvellet, Ryan Barber, Emmanuela Gaikedu, Simon Hay, Steve Lim, Chris Murray, David Pigott, Robert C. Reiner, Prasith Baccam, Heidi L. Gurung, Steven A. Stage, Bradley T. Suchoski, Chung-Yan Fong, Dit-Yan Yeung, Bijaya Adhikari, Jiaming Cui, B. Aditya Prakash, Alexander Rodríguez, Anika Tabassum, Jiajia Xie, John Asplund, Arden Baxter, Pinar Keskinocak, Buse Eylul Oruc, Nicoleta Serban, Sercan O. Arik, Mike Dusenberry, Arkady Epshteyn, Elli Kanal, Long T. Le, Chun-Liang Li, Tomas Pfister, Rajarishi Sinha, Thomas Tsai, Nate Yoder, Jinsung Yoon, Leyou Zhang, Daniel Wilson, Artur A. Belov, Carson C. Chow, Richard C. Gerkin, Osman N. Yogurtcu, Mark Ibrahim, Timothee Lacroix, Matthew Le, Jason Liao, Maximilian Nickel, Levent Sagun, Sam Abbott, Nikos I. Bosse, Sebastian Funk, Joel Hellewell, Sophie R. Meakin, Katharine Sherratt, Rahi Kalantari, Mingyuan Zhou, Morteza Karimzadeh, Benjamin Lucas, Thoai Ngo, Hamidreza Zoraghein, Behzad Vahedi, Zhongying Wang, Sen Pei, Jeffrey Shaman, Teresa K. Yamana, Dimitris Bertsimas, Michael L. Li, Saksham Soni, Hamza Tazi Bouardi, Madeline Adee, Turgay Ayer, Jagpreet Chhatwal, Ozden O. Dalgic, Mary A. Ladd, Benjamin P. Linas, Peter Mueller, Jade Xiao, Jurgen Bosch, Austin Wilson, Peter Zimmerman, Qinxia Wang, Yuanjia Wang, Shanghong Xie, Donglin Zeng, Jacob Bien, Logan Brooks, Alden Green, Addison J. Hu, Maria Jahja, Daniel McDonald, Balasubramanian Narasimhan, Collin Politsch, Samyak Rajanala, Aaron Rumack, Noah Simon, Ryan J. Tibshirani, Rob Tibshirani, Valerie Ventura, Larry Wasserman, John M. Drake, Eamon B. O’Dea, Yaser Abu-Mostafa, Rahil Bathwal, Nicholas A. Chang, Pavan Chitta, Anne Erickson, Sumit Goel, Jethin Gowda, Qixuan Jin, HyeongChan Jo, Juhyun Kim, Pranav Kulkarni, Samuel M. Lushtak, Ethan Mann, Max Popken, Connor Soohoo, Kushal Tirumala, Albert Tseng, Vignesh Varadarajan, Jagath Vytheeswaran, Christopher Wang, Akshay Yeluri, Dominic Yurk, Michael Zhang, Alexander Zlokapa, Robert Pagano, Chandini Jain, Vishal Tomar, Lam Ho, Huong Huynh, Quoc Tran, Velma K. Lopez, Jo W. Walker, Rachel B. Slayton, Michael A. Johansson, Matthew Biggerstaff, Nicholas G. Reich

**Affiliations:** 1 Department of Biostatistics and Epidemiology, University of Massachusetts Amherst, Amherst, MA 01003 USA; 2 Chair of Econometrics and Statistics, Karlsruhe Institute of Technology, Karlsruhe, 76185 Germany; 3 Computational Statistics Group, Heidelberg Institute for Theoretical Studies, Heidelberg, 69118 Germany; 4 IQT Labs, Waltham, MA 02451 USA; 5 Department of Statistics, Iowa State University, Ames, IA 50011 USA; 6 Institute of Stochastics, Karlsruhe Institute of Technology, Karlsruhe, Germany; 7 Institute of Mathematical Statistics and Actuarial Science, University of Bern, 3012 Bern, Switzerland; 8 Unaffiliated, New York, NY 10016 USA; 9 Walmart, Sunnyvale, CA 94086 USA; 10 Wadhwani Institute of Artificial Intelligence, Mumbai, Maharashtra 400093 India; 11 Biocomplexity Institute, University of Virginia, Charlottesville, Virginia 22904-4298 USA; 12 Department of Computer Science, University of Virginia, Charlottesville, Virginia 22904-4298 USA; 13 Discreet Labs, Raleigh, North Carolina USA; 14 Boston Children’s Hospital, Boston, Massachusetts, 02115 USA; 15 Harvard Medical School, Boston, Massachusetts, USA; 16 Texas Advanced Computing Center, Austin, Texas 78758 USA; 17 Department of Integrative Biology, University of Texas at Austin, Austin, TX 78712 USA; 18 Santa Fe Institute, Santa Fe, NM 87501 USA; 19 Department of Information, Risk, and Operations Management, University of Texas at Austin, Austin, TX 78712 USA; 20 Department of Statistics and Data Sciences, University of Texas at Austin, Austin, TX 78712 USA; 21 Ming Hsieh Department of Electrical and Computer Engineering, University of Southern California, Los Angeles, California 90089 USA; 22 Department of Computer Science, University of Southern California, Los Angeles, California 90089 USA; 23 US Army Engineer Research and Development Center, Concord, MA 01742 USA; 24 US Army Engineer Research and Development Center, Vicksburg, MS 39180 USA; 25 US Army Engineer Research and Development Center, Hanover, NH 03755 USA; 26 School of Medicine, State University of New York Upstate Medical University, Syracuse, NY 13210 USA; 27 Department of Psychiatry and Behavioral Sciences, State University of New York Upstate Medical University, Syracuse, NY 13210 USA; 28 Department of Public Health & Preventive Medicine, State University of New York Upstate Medical University, Syracuse, NY 13210 USA; 29 Department of Electrical Engineering and Computer Science, Syracuse University, Syracuse, NY 13210 USA; 30 Department of Physics, Trinity University, San Antonio, TX 78212 USA; 31 Department of Physics, University of Michigan - Ann Arbor, Ann Arbor, MI 48109 USA; 32 Departments of Epidemiology, Complex Systems, and Mathematics, University of Michigan - Ann Arbor, Ann Arbor, MI 48109 USA; 33 School of Public Health, University of Michigan - Ann Arbor, Ann Arbor, MI 48109 USA; 34 School of Public Health and Health Sciences, University of Massachusetts Amherst, Amherst, MA 01003 USA; 35 College of Information and Computer Sciences, University of Massachusetts Amherst, Amherst, MA 01003 USA; 36 Department of Statistics, University of Washington, Seattle, WA 98195 USA; 37 Halıcıoğlu Data Science Institute, University of California, San Diego, San Diego, CA 92093 USA; 38 Mechatronics, Embedded Systems and Automation Lab, Department of Mechanical Engineering, University of California Merced, Merced, CA 95301 USA; 39 Northeastern University, Boston, MA 02115 USA; 40 Department of Computer Science and Engineering, University of California, San Diego, San Diego, CA 93106 USA; 41 Department of Computer Science, University of California at Santa Barbara, Santa Barbara, CA 92093 USA; 42 Jilin University, Changchun City, Jilin Province PR China; 43 University of Science and Technology of China, Hefei, Anhui China; 44 Department of Computer Science, University of California, Los Angeles, CA USA; 45 Department of Medicine, University of Chicago, Chicago, IL 60637 USA; 46 University of Nebraska Omaha, Omaha, NE 68182 USA; 47 National Cancer Institute (NCI), NIH, Rockville, MD 20850 USA; 48 Department of Statistics and Data Science, University of Central Florida, Orlando, FL 32816 USA; 49 Department of Computer Science, University of Central Florida, Orlando, FL 32816 USA; 50 Department of Mathematics, University of Arizona, Tucson, AZ 85721 USA; 51 Department of Mechanical Engineering, Texas Tech University, Lubbock, Texas 79409 USA; 52 Construx Software, Bellevue, WA 98004 USA; 53 Construx, Bellevue, WA 98004 USA; 54 Quality Assurance and Data Science, Signature Science, LLC, Charlottesville, Virginia 22911 USA; 55 Quality Assurance and Data Science, Signature Science, LLC, Austin, Texas 78759 USA; 56 Swiss Data Science Center, EPFL & ETHZ, 1015 Lausanne, Switzerland; 57 Institute of Global Health, Faculty of Medicine, University of Geneva, 1202 Geneva, Switzerland; 58 Center for Intelligent Systems, EPFL, 1015 Lausanne, Switzerland; 59 Department of Civil and Environmental Engineering, University of Washington, Seattle, WA 98195 USA; 60 Department of Materials Science and Engineering, Rensselaer Polytechnic Institute, Troy, NY 12309 USA; 61 Unaffiliated, Davis, California, 95616 USA; 62 Brown University, Providence, RI 02912 USA; 63 School for Engineering of Matter, Transport and Energy, Arizona State University, Tempe, Arizona 85287 USA; 64 School of Engineering, Brown University, Providence, RI 02912 USA; 65 Infectious Disease Group, Predictive Science, Inc, San Diego, California, 92116 USA; 66 Department of Infectious Disease Epidemiology, Imperial College, London, Westminster, London W2 1PG UK; 67 University of Dallas, Irving, TX 75062 USA; 68 Unaffiliated, Seattle, WA USA; 69 Oliver Wyman Digital, Oliver Wyman, Boston, MA 02110 USA; 70 Oliver Wyman Digital, Oliver Wyman, Sao Paolo, 04711-904 Brazil; 71 Health & Life Sciences, Oliver Wyman, Boston, MA 2110 USA; 72 Financial Services, Oliver Wyman, New York, NY 10036 USA; 73 Oliver Wyman Digital, Oliver Wyman, New York, NY 10036 USA; 74 Health & Life Sciences, Oliver Wyman, New York, NY 10036 USA; 75 Core Consultant Group, Oliver Wyman, New York, NY 10036 USA; 76 Financial Services, Oliver Wyman, Toronto, ON M5J 0A1 Canada; 77 Financial Services, Oliver Wyman, Marylebone, London, W1U 8EW UK; 78 Department of Biological Sciences, University of Notre Dame, Notre Dame, IN 46556 USA; 79 Department of Industrial and Systems Engineering, North Carolina State University, Raleigh, NC 27695 USA; 80 Department of Mathematics and Statistics, Masaryk University, Brno, 61137 Czech Republic; 81 Microsoft, Redmond, WA 98029 USA; 82 Laboratory for the Modeling of Biological and Socio-technical Systems, Northeastern University, Boston, MA USA; 83 ISI Foundation, Turin, Italy; 84 Operations Research Center, Massachusetts Institute of Technology, Cambridge, MA 02139 USA; 85 Sloan School of Management, Massachusetts Institute of Technology, Cambridge, MA 02142 USA; 86 Leonard N Stern School of Business, New York University, NY USA; 87 Institute for Data, Systems, and Society, Massachusetts Institute of Technology, Cambridge, MA 02139 USA; 88 Laboratory for Computational Physiology, Massachusetts Institute of Technology, Cambridge, MA 02139 USA; 89 River Hill High School, Clarksville, MD USA; 90 Department of Computer Science and Engineering, Lehigh University, Bethlehem, PA 18015 USA; 91 Department of Industrial and Systems Engineering, Lehigh University, Bethlehem, PA 18015 USA; 92 College of Health, Lehigh University, Bethlehem, PA 18015 USA; 93 Department of Mathematics and Statistics, Northern Arizona University, Flagstaff, AZ 86011 USA; 94 Theoretical Division, Los Alamos National Laboratory, Los Alamos, NM 87545 USA; 95 Information Sciences Group, Los Alamos National Laboratory, Los Alamos, NM 87545 USA; 96 Theoretical Biology and Biophysics Group (T-6), Theoretical Division, Los Alamos National Laboratory, Los Alamos, NM 87545 USA; 97 Department of Biological Sciences, Northern Arizona University, Flagstaff, AZ 86011 USA; 98 Department of Chemistry and chemical biology, Cornell University, Ithaca, NY 14850 USA; 99 Life Sciences, JMP, LLC, Cary, NC 27513 USA; 100 Information Systems and Modeling Group, Los Alamos National Laboratory, Los Alamos, NM 87545 USA; 101 Statistical Sciences Group, Los Alamos National Laboratory, Los Alamos, NM 87545 USA; 102 Chair of Econometrics and Statistics, Karlsruhe Institute of Technology, Karlsruhe, Germany; 103 TRIUMF, Vancouver, BC V6T 2A3 Canada; 104 Department of Physics and Astronomy, University of Victoria, Victoria, BC V8W 2Y2 Canada; 105 Johns Hopkins University Applied Physics Lab, Laurel, MD 20723 USA; 106 Google Research, Mountainview, CA 94043 USA; 107 School of Engineering and Applied Sciences, Harvard University, Cambridge, MA 02134 US; 108 Department of Epidemiology, UNC Gillings School of Public Health, University of North Carolina at Chapel Hill, Chapel Hill, NC 27599 USA; 109 Department of Epidemiology, Harvard TH Chan School of Public Health, Boston, MA 02115 USA; 110 Department of Epidemiology, Johns Hopkins Bloomberg School of Public Health, Baltimore, MD 21205 USA; 111 Institute for Computational Medicine, Johns Hopkins University, Baltimore, MD 21218 USA; 112 Unaffiliated, Baltimore, MD 21205 USA; 113 Division of Epidemiology, Department of Internal Medicine, University of Utah, Salt Lake City, UT 84108 USA; 114 Laboratory of Ecohydrology, School of Architecture, Civil and Environmental Engineering, École Polytechnique Fédérale de Lausanne, Lausanne, 1015 Switzerland; 115 Department of Epidemiology, Gillings School of Global Public Health and The Carolina Population Center, University of North Carolina at Chapel Hill, Chapel Hill, NC 27599 US; 116 The Carolina Population Center, University of North Carolina at Chapel Hill, Chapel Hill, NC 27599 USA; 117 Unaffiliated, San Francisco, CA 94107 USA; 118 International Vaccine Access Center, Department of International Health, Department of Epidemiology, Johns Hopkins Bloomberg School of Public Health, Baltimore, MD 21231 USA; 119 Unaffiliated, San Francisco, CA 94122 USA; 120 Department of Civil and Systems Engineering, Johns Hopkins University, Baltimore, MD 21218-2682 USA; 121 Unaffiliated, Amsterdam, Netherlands; 122 Unaffiliated, Vienna, 1010 Austria; 123 Indiana University–Purdue University Indianapolis, Indianapolis, IN 46202 USA; 124 University of Illinois at Urbana-Champaign, Champaign, IL USA; 125 Analytics Center of Excellence, IQVIA, Plymouth Meeting, Pennsylvania, PA USA; 126 Analytics Center of Excellence, IQVIA, Cambridge, MA USA; 127 Georgia Institute of Technology, Atlanta, GA USA; 128 IQVIA, Evanston, IL USA; 129 University of Illinois at Urbana-Champaign, Champaign, IL USA; 130 Amplitude, San Francisco, CA USA; 131 Department of Finance, Iowa State University, Ames, IA 50011-1090 USA; 132 Department of Statistics, Iowa State University, Ames, IA 50011-1090 USA; 133 School of mathematical and statistical sciences, Clemson University, Clemson, SC 29634 USA; 134 Iowa State University, Ames, IA 50011-1091 USA; 135 Department of mathematics, College of William & Mary, Williamsburg, VA 23187 USA; 136 Department of Statistics, University of Virginia, Charlottesville, VA 22904 USA; 137 Institute of Business Forecasting (IBF), Great Neck, NY 11021 USA; 138 Imperial College London, London, UK; 139 Imperial College London, Brighton, UK; 140 University of Sussex, Falmer, Brighton, BN1 9RH UK; 141 Institute for Health Metrics and Evaluation, University of Washington, Seattle, WA 98121 USA; 142 Emerging Technologies, IEM, Inc, Bel Air, MD 21015 USA; 143 Emerging Technologies, IEM, Inc, Baton Rouge, LA 70809 USA; 144 The Hong Kong University of Science and Technology, Clear Water Bay, Hong Kong; 145 Department of Computer Science, University of Iowa, Iowa City, IA 52242 USA; 146 College of Computing, Georgia Institute of Technology, Atlanta, GA 30308 USA; 147 Georgia Institute of Technology, Atlanta, GA 30308 USA; 148 Department of Computer Science, Virginia Tech, Falls Church, VA 22043 USA; 149 Advanced Data Analytics, Metron, Inc, Reston, VA 20190 USA; 150 School of Industrial and Systems Engineering, Georgia Insitute of Technology, Atlanta, GA 30318 USA; 151 Google Cloud, Sunnyvale, CA 94089 USA; 152 Harvard University, Cambridge, MA 02138 USA; 153 Economic Research Department, Federal Reserve Bank of San Francisco, San Francisco, CA 94105 USA; 154 Office of Biostatistics and Epidemiology, Center for Biologics Evaluation and Research, Food and Drug Administration, Center for Biologics Evaluation and Research, Silver Spring, MD 20993 USA; 155 Mathematical Biology Section, NIDDK/LBM, NIH, Bethesda, MD 20892 USA; 156 School of Life Sciences, Arizona State University, Tempe, AZ 85287 USA; 157 Meta AI, New York, NY USA; 158 Meta AI, Paris, France; 159 Meta, Menlo Park, CA USA; 160 Centre for Mathematical Modelling of Infectious Diseases, London School of Hygiene & Tropical Medicine, London, UK; 161 London School of Hygiene & Tropical Medicine, London, UK; 162 Department of Electrical and Computer Engineering, The University of Texas at Austin, Austin, TX 78712 USA; 163 McCombs School of Business, The University of Texas at Austin, Austin, TX 78712 USA; 164 Department of Geography, Institute of Behavioral Science, University of Colorado Boulder, Boulder, CO 80309 USA; 165 Department of Geography, University of Colorado Boulder, Boulder, CO 80309 USA; 166 Social and Behavioral Science Research, Population Council, New York, NY 10017 USA; 167 Department of Environmental Health Sciences, Columbia University, New York, NY 10032 USA; 168 Radiology - Institute for Technology Assessment, Massachusetts General Hospital, Boston, MA 02114 USA; 169 Emory University Medical School, Atlanta, GA 30322 USA; 170 H. Milton Stewart School of Industrial and Systems Engineering, Georgia Institute of Technology, Atlanta, GA 30332 USA; 171 Harvard Medical School, Boston, MA 02114 USA; 172 Health Economic Modeling, Value Analytics Labs, Boston, MA 02114 USA; 173 Department of Medicine, Section of Infectious Diseases, Boston University School of Medicine, Boston, MA 02118 USA; 174 InterRayBio, LLC, Cleveland, Ohio, 44106 USA; 175 Center for Global Health & Diseases, Case Western Reserve University, Cleveland, OH 44106-4983 USA; 176 Department of Biostatistics, Columbia University, New York, NY 10032 USA; 177 Department of Biostatistics, UNC Chapel Hill, Chapel Hill, NC 27599 USA; 178 Marshall School of Business, Department of Data Sciences and Operations (DSO), University of Southern California, Los Angeles, CA 90089 USA; 179 Department of Statistics, Carnegie Mellon University, Pittsburgh, PA 15213 USA; 180 Department of Statistics, University of British Columbia, Vancouver, BC V6T 1Z4 Canada; 181 Department of Biomedical Data Sciences and Department of Statistics, Stanford University, Stanford, CA 94305-4020 USA; 182 Machine Learning Department, Carnegie Mellon University, Pittsburgh, PA 15213 USA; 183 Department of Statistics, Stanford University, Stanford, CA 94305-4020 USA; 184 Department of Biostatistics, University of Washington, Seattle, WA 98195 USA; 185 Center for the Ecology of Infectious Diseases, University of Georgia, Athens, GA 30602 USA; 186 California Institute of Technology, Pasadena, CA 91125 USA; 187 California Institute of Technology, Mountain View, CA 94043 USA; 188 California Institute of Technology, Chicago, IL 60606 USA; 189 California Institute of Technology, Redwood City, CA 94065 USA; 190 California Institute of Technology, Edison, NJ 08820 USA; 191 Center for Theoretical Physics, California Institute of Technology, Cambridge, MA 02139 USA; 192 Unaffiliated, Tucson, AZ 85710 USA; 193 Auquan, London, EC2A 4DP UK; 194 Auquan, Bengaluru, KA India; 195 Department of Mathematics and Statistics, Dalhousie University, Halifax, Nova Scotia B3H 4R2 Canada; 196 AIpert, San Carlos, CA 94070 USA; 197 Virtual Power System, Milpitas, CA 95035 USA; 198 Walmart Inc, Sunnyvale, CA 94085 USA; 199 Centers for Disease Control and Prevention, Atlanta, GA USA

**Keywords:** Databases, Viral infection, Software, Scientific data, Computer science

## Abstract

Academic researchers, government agencies, industry groups, and individuals have produced forecasts at an unprecedented scale during the COVID-19 pandemic. To leverage these forecasts, the United States Centers for Disease Control and Prevention (CDC) partnered with an academic research lab at the University of Massachusetts Amherst to create the US COVID-19 Forecast Hub. Launched in April 2020, the Forecast Hub is a dataset with point and probabilistic forecasts of incident cases, incident hospitalizations, incident deaths, and cumulative deaths due to COVID-19 at county, state, and national, levels in the United States. Included forecasts represent a variety of modeling approaches, data sources, and assumptions regarding the spread of COVID-19. The goal of this dataset is to establish a standardized and comparable set of short-term forecasts from modeling teams. These data can be used to develop ensemble models, communicate forecasts to the public, create visualizations, compare models, and inform policies regarding COVID-19 mitigation. These open-source data are available via download from GitHub, through an online API, and through R packages.

## Introduction

To understand how the COVID-19 pandemic would progress in the United States, dozens of academic research groups, government agencies, industry groups, and individuals produced probabilistic forecasts for COVID-19 outcomes starting in March 2020^1^. We collected forecasts from over 90 modeling teams in a data repository, thus making forecasts easily accessible for COVID-19 response efforts and forecast evaluation. The data repository is called the US COVID-19 Forecast Hub (hereafter, Forecast Hub) and was created through a partnership between the United States Centers for Disease Control and Prevention (CDC) and an academic research lab at the University of Massachusetts Amherst.

**Fig. 1:**
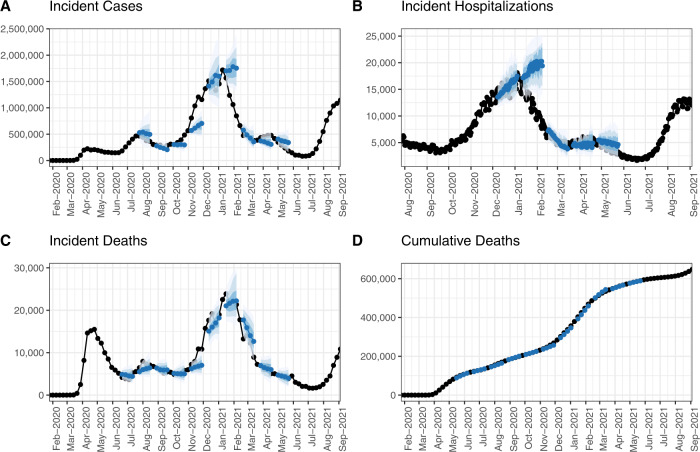
Time series of weekly incident deaths at the national level and forecasts from the COVID-19 Forecast Hub ensemble model for selected weeks in 2020 and 2021. Ensemble forecasts (blue) with 50%, 80% and 95% prediction intervals shown in shaded regions and the ground-truth data (black) for incident cases (**A**), incident hospitalizations (**B**), incident deaths (**C**) and cumulative deaths (**D**). The truth data come from JHU CSSE (panels **A**, **C**, **D**) and HealthData.gov (panel **B**).

The Forecast Hub was launched in early April 2020 and contains real-time forecasts of reported COVID-19 cases, hospitalizations, and deaths. As of May 3^rd^, 2022, the Forecast Hub had collected over 92 million individual point or quantile predictions contained within over 6,600 submitted forecast files from 110 unique models. The forecasts submitted each week reflected a variety of forecasting approaches, data sources, and underlying assumptions. There were no restrictions in place regarding the underlying information or code used to generate real-time forecasts. Each week, the latest forecasts were combined into an ensemble forecast (Fig. 1), and all recent forecast data were updated on an official COVID-19 Forecasting page hosted by the US CDC (https://www.cdc.gov/coronavirus/2019-ncov/science/forecasting/mathematical-modeling.html). The ensemble models were also used in the weekly reports that are posted on the Forecast Hub website, https://covid19forecasthub.org/doc/reports/.

Forecasts are quantitative predictions about data that will be observed at a future time. Forecasts differ from scenario-based projections, which examine feasible outcomes conditional on a variety of future assumptions. Because forecasts are unconditional estimates of data that will be observed in the future, they can be evaluated against eventual observed data. An important feature of the Forecast Hub is that submitted forecasts are time-stamped so the exact time at which a forecast was made public can be verified. In this way, the Forecast Hub serves as a public, independent registration system for these forecast model outputs. Data from the Forecast Hub have served as the basis for research articles for forecast evaluation^2^ and forecast combination^3–5^. These studies can be used to determine how well models have performed at various points during the pandemic, which can, in turn, guide best practices for utilizing forecasts in practice and inform future forecasting efforts^2^.

Teams submitted predictions in a structured format to facilitate data validation, storage, and analysis. Teams also submitted a metadata file and license for their model’s data. Forecast data, ground truth data from the Johns Hopkins University Center for Systems Science and Engineering (JHU CSSE)^6^, New York Times (NYTimes)^7^, USA Facts^8^, and HealthData.gov^9^ and model metadata were stored in the public Forecast Hub GitHub repository^10^.

**Fig. 2:**
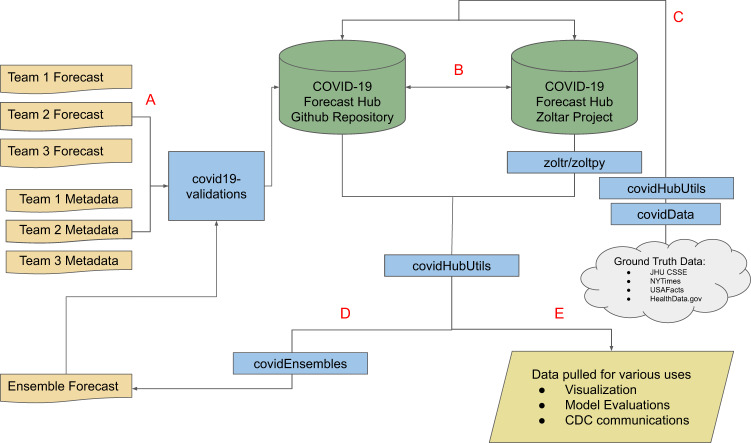
Schematic of the data storage and related infrastructure surrounding the COVID-19 Forecast Hub. (**A**) Forecasts are submitted to the COVID-19 Forecast Hub GitHub repository and undergo data format validation before being accepted into the system. (**B**) A continuous integration service ensures that the GitHub repository and PostgreSQL database stay in sync with mirrored versions of the data. (**C**) Truth data for visualization, evaluation, and ensemble building are retrieved once per week using both the *covidHubUtils* and the *covidData* R packages. Truth data are stored in both repositories. (**D**) Once per week, an ensemble forecast submission is made using the *covidEnsembles* R package. It is submitted to the GitHub repository and undergoes the same validation as other submissions. (**E**) Using the *covidHubUtils* R package, forecast and truth data may be extracted from either the GitHub or PostgreSQL database in a standard format for tasks such as scoring or plotting.

The forecasts were automatically synchronized with an online database called Zoltar via calls to a representational State Transfer (REST) application programming interface (API)^11^ every six hours (Fig. 2). Raw forecast data may be downloaded directly from GitHub or Zoltar via the *covidHubUtils* R package^12^, the *zoltr* R package^13^ or *zoltpy* Python library^14^.

This dataset of real-time forecasts created during the COVID-19 pandemic can provide insights into the shortcomings and successes of predictions and improve forecasting efforts in years to come. Although these data are restricted to forecasts for COVID-19 in the United States, the structure of this dataset has been used to create datasets of COVID-19 forecasts in the EU and the UK, and longer-term scenario projections in the US^15–18^. The general structure of this data collection could be applied to additional diseases or forecasting outcomes in the future^11^.

This large collaborative effort has provided data on short-term forecasts for over two years of forecasting efforts. Nearly all data were collected in real time and therefore are not subject to retrospective biases. The data are also openly available to the public, thus fostering a transparent, open science approach to support public health efforts.

## Results

### Data acquisition

Beginning in April 2020, the Reich Lab at the University of Massachusetts, Amherst, in partnership with the US CDC, began collecting probabilistic forecasts of key COVID-19 outcomes in the United States (Table 1). The effort began by collecting forecasts of deaths and hospitalizations at the weekly and daily scales for the 50 US states and 5 territories (Washington DC, Puerto Rico, US Virgin Islands, Guam, and the Northern Mariana Islands) as well as the aggregated US national level. In July 2020, daily resolution-level forecasts for COVID-19 deaths were discontinued, and the effort expanded to include forecasts of weekly incident cases at the county, state, and national levels. Forecasts may include a point prediction and/or quantiles of a predictive distribution.

**Table 1:** Forecast characteristics for all four outcomes.

Outcome	Scale	Locations			Horizons Stored	Number of quantiles for probabilistic forecasts	Earliest Forecast Date	First date of standardized truth data	Date of first ensemble forecast
		County	State	National					
Incident Cases	Weekly	X	X	X	1 - 8 weeks	7	2020-07-05	2020-03-15	2020-07-18
Incident Hospitalizations	Daily		X	X	1 - 130 days	23	2020-03-27	2020-11-16	2020-12-05
Incident Deaths	Daily		X	X	1 - 130 days	23	2020-03-15	2020-03-15	NA
Incident Deaths	Weekly		X	X	1-20 weeks	23	2020-03-15	2020-03-15	2020-06-20
Cumulative Deaths	Daily		X	X	1 - 130 days	23	2020-03-15	2020-03-15	NA
Cumulative Deaths	Weekly		X	X	1-20 weeks	23	2020-03-15	2020-03-15	2020-04-13

The table shows the temporal scale, spatial scale of locations, horizons stored, number of quantiles, and dates of the earliest forecast, earliest standardized truth data, and earliest ensemble build.

Any team was eligible to submit data to the Forecast Hub provided they used the correct formatting. Upon initial submission of forecast data, teams were required to upload a metadata file that briefly described the methods used to create the forecasts and specified a license under which their forecast data were released. Individual model outputs are available under different licenses as specified in the GitHub data repository. No model code was stored in the Forecast Hub.

During the first month of operation, members of the Forecast Hub team downloaded forecasts made available by teams publicly online, transformed these forecasts into the correct format (see *Forecast format* section), and pushed them into the Forecast Hub repository. Starting in May 2020, all teams were required to format and submit their own forecasts.

### Repository structure

The dataset containing forecasts is stored in two locations, and all data can be accessed through either source. The first is the COVID-19 Forecast Hub GitHub repository, https://github.com/reichlab/covid19-forecast-hub, and the second is an online database, Zoltar, which can be accessed via a REST API^11^. Details about data access and format are documented in the subsequent sections.

When accessing data through the Zoltar forecast repository REST API, subsets of submitted forecasts can be queried directly from a PostgreSQL database. This eliminates the need to access individual CSV files and facilitates access to versions of forecasts in cases when they were updated.

### Forecast outcomes

The Forecast Hub dataset stores forecasts for four different outcomes: incident cases, incident hospitalizations, incident deaths, and cumulative deaths (Table 1). Incident case forecasts were first introduced as a forecast outcome several months after the Forecast Hub started and have several key differences from other predicted outcomes. They are the only outcomes for which the Forecast Hub accepts county-level forecasts, as well as state and national level forecasts. Since there are over 3,000 counties in the US, this required some compromises on the scale of data collected for these forecasts in other ways. Specifically, case forecasts may only be submitted for up to 8 weeks into the future instead of up to 20 weeks for deaths and are required to have fewer quantiles (seven quantiles) compared to other outcomes, which can have up to twenty-three quantiles. This gives a coarser representation of the forecast (see the section on Forecast format below).

### Forecast target dates

Weekly targets follow the standard of epidemiological weeks (EW) used by the CDC, which defines a week as starting on Sunday and ending on the following Saturday^19^. Forecasts of cumulative deaths target the number of cumulative deaths reported by Saturday ending a given week. Forecasts of weekly incident cases or deaths target the difference between reported cumulative cases or deaths on consecutive Saturdays. As an example of a forecast and the corresponding observation, forecasts submitted between Tuesday, October 6, 2020 (day 3 of EW41) and Monday, October 12, 2020 (day 2 of EW42) contained a “1 week ahead” forecast of incident deaths that corresponded to the change in cumulative reported deaths observed in EW42 (i.e., the difference between the cumulative reported deaths on Saturday, October 17, 2020, and Saturday, October 10, 2020), a “2 week ahead” forecast that corresponded to the change in cumulative reported deaths in week EW43. In this paper, we refer to the “forecast week” of a submitted forecast as the week corresponding to a “0-week ahead” horizon. In the example above, the forecast week would be EW41. Daily incident hospitalization horizons are for the number of reported hospitalizations a specified number of days after the forecast was generated.

### Summary of forecast data collected

**Fig. 3:**
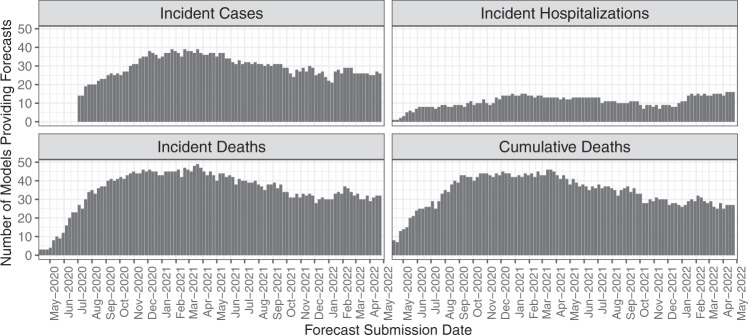
Number of primary forecasts submitted for each outcome per week from April 27^th^, 2020 through May 3^rd^, 2022. In the initial weeks of submission, fewer than 10 models provided forecasts. Over time, the number of teams submitting forecasts for each forecasted outcome increased into early 2021 and then saw a small decline through the end of 2021, with some renewed interest in 2022.

In the initial weeks of submission, fewer than 10 models provided forecasts. As the pandemic spread, the number of teams submitting forecasts increased; as of May 3^rd^, 2022, 93 primary, 9 secondary models, and 17 models with the designation “other” had been submitted to the Forecast Hub. As of May 3^rd^, 2022, across all weeks, a median of 30 primary models (range: 14 to 39) contributed incident case forecasts (Fig. 3a), a median of 11 primary models (range: 1 to 16) contributed incident hospitalizations (Fig. 3b), a median of 37 primary models (range 1 to 49) contributed incident death forecasts (Fig. 3c), and a median of 35 primary models (range 3 to 46) contributed cumulative death forecasts each week (Fig. 3d). As of May 3^rd^, 2022, the dataset contained 6,633 forecast files with 92,426,015 point or quantile predictions for unique combinations of targets and locations.

### Ensemble and baseline forecasts

Alongside the models submitted by individual teams, there are also baseline and ensemble models generated by the Forecast Hub and CDC.

The COVIDhub-baseline model was created by the Forecast Hub in May 2020 as a benchmarking model. Its point forecast is the most recent observed value as of the forecast creation date with a probability distribution around that based on weekly differences in previous observations^2^. The baseline model initially produced forecasts for case and death outcomes. Hospitalization baseline forecasts were added in September 2021.

The COVIDhub-ensemble model creates a combination of submitted forecasts to the Forecast Hub. The ensemble produces forecasts of incident cases at a horizon of 1 week ahead, forecasts of incident hospitalizations at horizons up to 14 days ahead, and forecasts of incident and cumulative deaths at horizons up to 4 weeks ahead. Initially the ensemble produced forecasts of incident cases at horizons of 1 to 4 weeks and incident hospitalizations at 1 to 28 days. However, in September 2021, due to the unreliability of incident case and hospitalization forecasts at horizons greater than 1 week (for cases) and 14 days (for hospitalizations), horizons past those respective thresholds were excluded from the COVIDhub-ensemble model, although they were still included in the COVIDhub-4_week_ensemble^20^. Other work details the methods used for determining the appropriate combination approach^3,4^. Starting in February 2021, GitHub tags were created to document the exact version of the repository used each week to create the COVIDhub-ensemble forecast. This creates an auditable trail in the repository so the correct version of the forecasts used could be recovered even in cases when some forecasts were subsequently updated.

The Forecast Hub also collaborates with the CDC on the production of three additional ensemble forecasts each week. These are the COVIDhub-4_week_ensemble, COVIDhub-trained_ensemble, and the COVIDhub_CDC-ensemble. The COVIDhub-4_week_ensemble produces forecasts of incident cases, incident deaths, and cumulative deaths at horizons of 1 through 4 weeks ahead, and forecasts of incident hospitalizations at horizons of 1 through 28 days ahead and uses the equally-weighted median of all component forecasts at each location, forecast horizon, and quantile level. The COVIDhub-trained_ensemble uses the same targets as the COVIDhub-4_week_ensemble but computes the models as a weighted median of the ten component forecasts with the best performance as measured by their weighted interval score (WIS) in the 12 weeks prior to the forecast date. The COVIDhub_CDC-ensemble pulls forecasts of cases and hospitalizations from the COVIDhub-4_week_ensemble and forecasts of deaths from the COVIDhub-trained_ensemble. The set of horizons that are included is updated regularly using rules developed by the CDC based on recent forecast performance.

Several other models are also combinations of some or all models submitted to the Forecast Hub. As of May 3^rd^, 2022, these models are FDANIHASU-Sweight, JHUAPL-SLPHospEns, and KITmetricslab-select_ensemble. These models are flagged in the metadata using the Boolean metadata field, “ensemble_of_hub_models”.

### Use scenarios

#### R package covidHubUtils

We have developed the *covidHubUtils* R package at https://github.com/reichlab/covidHubUtils to facilitate bulk retrieval of forecasts for analysis and evaluation. Examples of how to use the *covidHubUtils* package and its functions can be found at https://reichlab.io/covidHubUtils/. The package supports loading forecasts from a local clone of the GitHub repository or by querying data from Zoltar. The package supports common actions for working with the data, such as loading specific subsets of forecasts, plotting forecasts, scoring forecasts, retrieving ground truth data, and many other utility functions to simplify working with the data.

#### Visualization of forecasts in the COVID-19 Forecast Hub

**Fig. 4:**
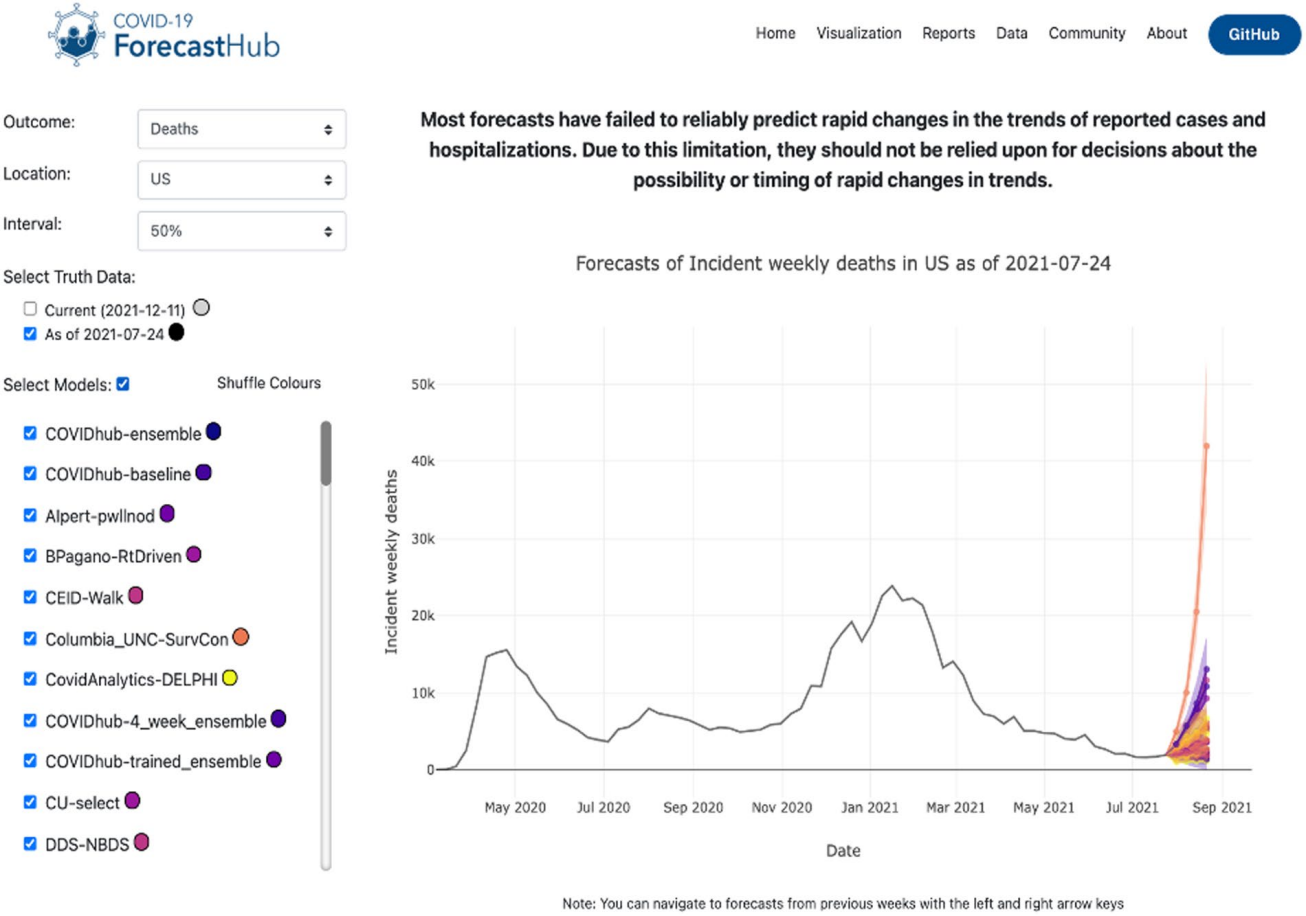
Visualization tool updated weekly by the US COVID-19 Forecast Hub displays model forecasts and truth data at selected forecast dates, locations, forecast outcomes and PI levels. US national level incident death forecasts from 39 models are shown with point values and a 50% PI. These forecasts are for 1 through 4 week ahead horizons. Data used for forecasting were generated on July 24th, 2021. The visualization tool is available at: https://viz.covid19forecasthub.org.

In addition to viewing forecasts in an R package, forecasts can also be viewed through our public website, https://viz.covid19forecasthub.org/. Through this tool, viewers can select the outcome, location, prediction interval, issue date of the truth data, and the models of interest to view forecasts. This tool can be used to see forecasts for the upcoming weeks, qualitatively evaluate model performance in past weeks, or visualize past performance based on available data at the time of forecasting (Fig. 4).

#### Communicating results from the COVID-19 Forecast Hub

Communication of probabilistic forecasts to the public is challenging^21,22^, and the best practices regarding the communication of outbreaks are still developing^23^. Starting in April 2020, the CDC published weekly summaries of these forecasts on their public website^24^, and these forecasts were occasionally used in public briefings by the CDC Director^25^. Additional examples of the communication of Forecast Hub data can be viewed through weekly reports generated by the Forecast Hub team for dissemination to the general public, including state and local departments of health(https://covid19forecasthub.org/doc/reports/). On December 22nd, 2021, the CDC ceased communication of case forecasts due to low reliability of these forecasts (https://www.cdc.gov/coronavirus/2019-ncov/science/forecasting/forecasts-cases.html).

## Discussion

We present here the US COVID-19 Forecast Hub, a data repository that stores structured forecasts of COVID-19 cases, hospitalizations, and deaths in the United States. The Forecast Hub is an important asset for visualizing, evaluating, and generating aggregate forecasts. It also demonstrates the highly collaborative effort that has gone into COVID-19 modeling efforts. This open-source data repository is beneficial for researchers, modelers, and casual viewers interested in forecasts of COVID-19. The website was viewed over half a million times in the first two years of the pandemic.

The US COVID-19 Forecast Hub is a unique, large-scale, collaborative infectious disease modeling effort. The Forecast Hub emerged from years of collaborative modeling efforts that started as government sponsored forecasting “challenges”. These collaborations are distinct from modeling efforts of individual teams, as the Forecast Hub has created open collaborative systems that facilitate model collection, curation, comparison, and combination, often in direct collaboration with governmental public health agencies^26–28^. The Forecast Hub built on these past efforts by developing a new quantile-based data format as well as automated data submission and validation procedures. Additionally, the scale of the collaborative effort for the US COVID-19 Forecast Hub has exceeded prior COVID-19 forecasting efforts by an order of magnitude in terms of the number of participating teams and forecasts collected. Finally, the infrastructure developed for the US COVID-19 Forecast Hub has been adapted for use by a number of other modeling hubs, including the US COVID-19 Scenario Modeling Hub^17^, the European COVID-19 Forecast Hub^15^, the German/Polish COVID-19 Forecasting Hub^16^, the German COVID-19 Hospitalization Nowcasting Hub^29^, and the 2022 US CDC Influenza Hospitalization Forecasting challenge^30^.

The Forecast Hub has played a critical role in collecting forecasts in a single format from over 100 different prediction models and making these data available to a wide variety of stakeholders during the COVID-19 pandemic. While some of these teams register their forecasts in other publicly available locations, many teams do not. Thus the Forecast Hub is the only location where many teams’ forecasts are available. In addition to curating data from other models, the Forecast Hub has also played a central role in synthesizing the outputs of models together. The Forecast Hub has generated an ensemble forecast, which has been used in official communications by the CDC, every week since April 2020. The ensemble model for incident deaths, a median aggregate of all other eligible models, was consistently the most accurate model when aggregated across forecast targets, weeks, and locations, even though it was rarely the single most accurate forecast for any single prediction^2^.

The US COVID-19 Forecast Hub has built a specific set of open-source tools that have facilitated the development of operational stand-alone and ensemble forecasts for the pandemic. However, the structure of the tools is quite general and could be adapted for use in other real-time prediction efforts. Additionally, the Forecast Hub infrastructure and data described represent best practices for collecting, aggregating, and disseminating forecasts^31^. The US COVID-19 Forecast Hub has developed and operationalized one standardized forecast format, time-stamped submissions, open access, and a collection of tools to facilitate working with the data.

The data in this hub will be useful in the future for continuing analysis and comparisons of forecasting methods. The data can also be used as an exploratory dataset for creating and testing novel models and methods for model analysis (e.g., new ways to create an ensemble or post hoc forecast calibration methods). Because the data serve as an open repository of the state of the art in infectious disease forecasting, they will also be helpful as a retrospective reference point for comparison when new forecasting models are developed.

Model coordination efforts occur in many fields –including climate science^32^, ecology^33^, and space weather^34^, among others– to inform policy decisions by curating many models and synthesizing their outputs and uncertainties. Such efforts ensure that individual model outputs may indeed be easily compared to and assimilated with one another, and thus play a role in making scientific research more rigorous and transparent. As the use of advanced computational models becomes more commonplace in a wide range of scientific fields, model coordination projects and model output standardization efforts will play an increasingly important role in ensuring that policy makers can be provided with a unified set of model outputs.

## Methods

### Forecast assumptions

Forecasters used a variety of assumptions to build models and generate predictions. Forecasting approaches include statistical or machine learning models, mechanistic models incorporating disease transmission dynamics, and combinations of multiple approaches^2^. Teams have also included varying assumptions regarding future changes in policies and social distancing measures, the transmissibility of COVID-19, vaccination rates, and the spread of new virus variants throughout the United States.

### Weekly submissions

A forecast submission consists of a single comma-separated value (CSV) file submitted via pull request to the GitHub repository. Forecast submissions are validated for technical accuracy and formatting (see below) using automated checks implemented by continuous integration servers before being merged. To be included in the weekly ensemble model, teams were required to submit their forecast on Sunday or prior to a deadline on Monday. The majority of teams contributing to the dataset submitted forecasts to the Forecast Hub repository on Sunday or Monday, although some teams submitted at other times depending on their model production schedule.

### Exclusion criteria

No forecasts were excluded from the dataset due to the forecast values or the background experience of the forecasters. Forecast files were only rejected if they did not meet the automatic formatting criteria implemented through automatic GitHub checks^35^. These included checks to ensure that, among other criteria:

- A forecast file is submitted no more than two days after it has been created (to ensure forecasts submitted were truly prospective). The creation date is based on the date in the filename created by the submitting team.
- The forecast dates in the content of the file are in the format YYYY-MM-DD and must match the creation date.
- Quantile forecasts do not contain any quantiles at probability levels other than the required levels (see Forecast Format section below).

### Updates to files

To ensure that forecasting is done in real-time, all forecasts are required to be submitted to the Forecast Hub within 2 days of the forecast date, which is listed in a column within each forecast file. Although occasional late submissions were accepted through January 2021, the policy was updated to not accept late forecasts due to missed deadlines, updated modeling methods, or other reasons.

Exceptions to this policy were made if there was a bug that affected the forecasts in the original submission or if a new team joined. If there was a bug, teams were required to submit a comment with their updated submission affirming that there was a bug and that the forecast was only produced using data that were available at the time of the original submission. In the case of updates to forecast data, both the old and updated versions of the forecasts can be accessed either through the GitHub commit history or through time-stamped queries of the forecasts in the Zoltar database. Note that an updated forecast can include “retracting” a particular set of predictions in the case when an initial forecast was not able to be updated. When new teams join the Forecast Hub, they can submit late forecasts if they can provide publicly available evidence that the forecasts were made in real-time (e.g., GitHub commit history).

### Ground truth data

Data from the JHU CSSE dataset^36^ are used as the ground truth data for cases and deaths. Data from the HealthData.gov system for state-level hospitalizations are used for the hospitalization outcome. JHU CSSE obtained counts of cases and deaths by collecting and aggregating reports from state and local health departments. HealthData.gov contains reports of hospitalizations assembled by the U.S. Department of Health and Human Services. Teams were encouraged to use these sources to build models. Although hospitalization forecasts were collected starting in March 2020, hospitalization data from HealthData.gov were only available later, and we started encouraging teams to target these data in November 2020. Some teams used alternate data sources, including the NYTimes, USAFacts, US Census data, and other signals^2^. Versions of truth data from JHU CSSE, USAFacts, and the NYTimes are stored in the GitHub repository.

Previous reports of ground truth data for past time points were occasionally updated as new records became available, definitions of reportable cases, deaths, or hospitalizations changed, or errors in data collection were identified and corrected. These revisions to the data are sometimes quite substantial^35,36^, and for purposes such as retrospective ensemble construction, it is necessary to use the data that would have been available in real-time. The historically versioned data can be accessed either through GitHub commit records, data versions released on HealthData.gov, or third-party tools such as the covidcast API provided by the Delphi group at Carnegie Mellon University or the *covidData* R package^37^.

### Model designation

Each model stored in the repository must have a classification of “primary,” “secondary”, or “other”. Each team must only have one “primary” model. Teams submitting multiple models with similar forecasting approaches can use the designations “secondary” or “other” for their models. Models with the designation “primary” are included in evaluations, the weekly ensemble, and the visualization. The “secondary” label is designed for models that have a substantive methodological difference than a team’s “primary” model. Models with the designation “secondary” are included only in the ensemble and the visualization. The “other” label is designed for models that are small variations on a team’s “primary” model. Models with the designation “other” are not included in evaluations, the ensemble build, or the visualization.

### GitHub repository data structure

Forecasts in the GitHub repository are available in subfolders organized by model. Folders are named with a team name and model name, and each folder includes a metadata file and forecast files. Forecast CSV files are named using the format “<YYYY-MM-DD>-<team abbreviation>-<model abbreviation>.csv”. In these files, each row contains data for a single outcome, location, horizon, and point or quantile prediction as described above.

The metadata file for each team, named using the format “metadata-<team abbreviation>-<model abbreviation>.txt”, contains relevant information about the team and the model that the team is using to generate forecasts.

### Forecast format

Forecasts were required to be submitted in the format of point predictions and/or quantile predictions. Point predictions represented single “best” predictions with no uncertainty, typically representing a mean or median prediction from the model. Quantile predictions are an efficient format for storing predictive distributions of a wide range of outcomes.

Quantile representations of predictive distributions lend themselves to natural computations of, for example, pinball loss or a weighted interval score, both proper scoring rules that can be used to evaluate forecasts^38^. However, they do not capture the structure of the tails of the predictive distribution beyond the reported quantiles. Additionally, the quantile format does not preserve any information on correlation structures between different outcomes.

The forecast data in this dataset are stored in seven columns:

1. **forecast_date** - the date the forecast was made in the format YYYY-MM-DD.
2. **target** - a character string giving the number of days/weeks ahead that are being forecasted (horizon) and the outcome. Horizons must be one of the following:

- “N wk ahead cum death” where N is a number between 1 and 20
- “N wk ahead inc death” where N is a number between 1 and 20
- “N wk ahead inc case” where N is a number between 1 and 8
- “N day ahead inc hosp” where N is a number between 0 and 130

3. **target_end_date** - a character string representing the date for the forecast target in the format YYYY-MM-DD. For “k day-ahead” targets, target_end_date will be k days after forecast_date. For “k week ahead” targets, target_end_date will be the Saturday at the end of the specified epidemic week, as described above.
4. **location** - character string of Federal Information Processing Standard Publication (FIPS) codes identifying U.S. states, counties, territories, and districts as well as “US” for national forecasts. The values for the FIPS codes are available in a CSV file in the repository and as a data object in the covidHubUtils R package for convenience.
5. **type** - character value of “point” or “quantile” indicating whether the row corresponds to a point forecast or a quantile forecast.
6. **quantile** - the probability level for a quantile forecast. For death and hospitalization forecasts, forecasters can submit quantiles at 23 probability levels: 0.01, 0.025, 0.05, 0.10, 0.15…, 0.95, 0.975, and 0.99. For cases, teams can submit up to 7 quantiles at levels .025, 0.100, 0.250, 0.5, 0.750, 0.900 and 0.975. If the forecast “type” is equal to “point”, the value in the quantile column is equal to “NA”.
7. **value** – non-negative numbers indicating the “point” or “quantile” prediction for the row. For a “point” prediction, the value is simply the value of that point prediction for the target and location associated with that row. For a “quantile” prediction, the model predicts that the eventual observation will be less than or equal to this value with the probability given by the quantile probability level.

### Metadata format

Each team documents their model information in a metadata file which is required along with the first forecast submission. Each team is asked to record their model’s design and assumptions, the model contributors, the team’s website, information regarding the team’s data sources, and a brief model description. Teams may update their metadata file periodically to keep track of minor changes to a model.

A standard metadata file should be a YAML file with the following required fields in a specific order:

1. **team_name** - the name of the team (less than 50 characters).
2. **model_name** - the name of the model (less than 50 characters).
3. **model_abbr** - an abbreviated and uniquely identified name for the model that is less than 30 alphanumeric characters. The model abbreviation must be in the format of ‘[team_abbr]-[model_abbr]‘ where each of the ‘[team_abbr]‘ and ‘[model_abbr]‘ are text strings that are each less than 15 alphanumeric characters that do not include a hyphen or whitespace.
4. **model_contributors** - a list of all individuals involved in the forecasting effort, affiliations, and email addresses. At least one contributor needs to have a valid email address. The syntax of this field should be name1 (affiliation1) <user@address>, name2 (affiliation2) <user2@address2>
5. website_url* - a URL to a website that has additional data about the model. We encourage teams to submit the most user-friendly version of the model, e.g., a dashboard, or similar, that displays the model forecasts. If there is an additional data repository where forecasts and other model code are stored, this can be included in the methods section. If only a more technical site, e.g., GitHub repo, exists, that link should be included here.
6. **license** - one of the acceptable license types in the Forecast Hub. We encourage teams to submit as a “cc-by-4.0” to allow the broadest possible use, including private vaccine production (which would be excluded by the “cc-by-nc-4.0” license). If the value is “LICENSE.txt”, then a LICENSE.txt file must exist within the model folder and provide a license.
7. **team_model_designation** - upon initial submission this field should be one of “primary”, “secondary” or “other”.
8. **methods** - a brief description of the forecasting methodology that is less than 200 Characters.
9. **ensemble_of_hub_models** - a Boolean value (‘true‘ or ‘false‘) that indicates whether a model combines multiple hub models into an ensemble.

*in earlier versions of the metadata files, this field was named **model_output**.

Teams are also encouraged to add model information with optional fields described below:

1. **institution_affil** - University or company names, if relevant.
2. **team_funding** - Like an acknowledgement in a manuscript, teams can acknowledge funding here.
3. **repo_url** - A GitHub repository url or something similar.
4. **twitter_handles** - one or more Twitter handles (without the @) separated by commas.
5. **data_inputs** - A description of the data sources used to inform the model and the truth data targeted by model forecasts. Common data sources are NYTimes, JHU CSSE, COVIDTracking, Google mobility, HHS hospitalization, etc. An example description could be “case forecasts use NYTimes data and target JHU CSSE truth data, hospitalization forecasts use and target HHS hospitalization data”
6. **citation** - a url (doi link preferred) to an extended description of the model, e.g., blog post, website, preprint, or peer-reviewed manuscript.
7. **methods_long** - An extended description of the methods used in the model. If the model is modified, this field can be used to provide the date of the modification and a description of the change.

## Technical Validations

Two similar but distinct validation processes were used to validate data on the GitHub repository and on Zoltar.

### Validations during data submission

Validations were set up using GitHub Actions to manage the continuous integration and automated data checking^35^. Teams submitted their metadata files and forecasts through pull requests on GitHub. Each time a new pull request was submitted, a validation script ran on all new or updated files in the pull request to test for their validity. Separate checks ran on metadata file changes and forecast data file changes.

The metadata file for each team was required to be in a valid YAML format, and a set of specific checks were required before a new metadata file could be merged into the repository. Checks included ensuring that all metadata files are using the rules outlined in the Metadata Format section, that the proposed team and model names do not conflict with existing names, that a valid license for data reuse is specified, and that a valid model designation was present. Additionally, each team must have their files under a folder named consistently with their *model_abbr*, and they must only have one *primary* model.

New or changed forecast data files for each team were required to pass a series of checks for data formatting and validity. These checks also ensured that the forecast data files did not meet any of the exclusion criteria (see the Methods section for specific rules). Each forecast file is subject to the validation rules documented at: https://github.com/reichlab/covid19-forecast-hub/wiki/Forecast-Checks.

### Validations on Zoltar

When a new forecast file is uploaded to Zoltar, unit tests are run on the file to ensure that forecast elements contain a valid structure. (For a detailed specification of the structure of forecast elements, see https://docs.zoltardata.com/validation/.) If a forecast file does not pass all unit tests, the upload will fail and the forecast file will not be added to the database; only when all tests pass will the new forecast be added to Zoltar. The validations in place on GitHub ensure that only valid forecasts will be uploaded to Zoltar.

### Truth data

Raw truth data from multiple sources including JHU, NYTimes, USAFacts, and Healthdata.gov, were downloaded and reformatted using the scripts in the R packages *covidHubUtils* (https://github.com/reichlab/covidHubUtils) and *covidData* (https://github.com/reichlab/covidData. This data generating process is automated by GitHub Actions every week, and the results (called “truth data”) are directly uploaded to the Forecast Hub repository and Zoltar. Specifically, case and death raw truth data were aggregated to a weekly level, and all three outcomes (cases, deaths, and hospitalization) are reformatted for use within the Forecast Hub.

## Data Availability

The datasets generated and/or analyzed during the current study are available in the reichlab/covid19-forecast-hub GitHub repository, https://github.com/reichlab/covid19-forecast-hub. A permanent DOI for the GitHub repository for the Forecast Hub is available as 10.5281/zenodo.5208210^10^ Forecast data are also available through our Zoltar forecast repository at https://zoltardata.com/project/44.

## Code availability

All code for forecast data validation and storage associated with the current submission is available in the Forecast Hub GitHub repository, https://github.com/reichlab/covid19-forecast-hub-validations. Ensemble models are built with code in the *covidEnsembles* R package, https://github.com/reichlab/covidEnsembles. The code for forecast analysis is at 10.5281/zenodo.5207940^12^ (covidHubUtils R package) and 10.5281/zenodo.5208224^7^ (covidData R package). Any updates will also be published on Zenodo.
